# THE DAILY BALANCE PROJECT: AN INQUIRY INTO THE INTRAINDIVIDUAL DYNAMICS OF PERCEIVED AND ACTUAL FALL RISK

**DOI:** 10.1093/geroni/igz038.2289

**Published:** 2019-11-08

**Authors:** Shannon T Mejia, Katherine Hsieh, Jason Fanning, Jacob Sosnoff

**Affiliations:** 1 University of Illinois, Urbana-Champaign, Champaign, Illinois, United States; 2 University of Illinois, Urbana-Champaign, Urbana, Illinois, United States; 3 Wake Forest University, Winston-Salem, Illinois, United States

## Abstract

An accurate understanding of one’s abilities and limitations allows adaptive response to the challenges that are faced in daily life. However, older adults may over or under estimate their actual abilities. The Daily Balance Project examined the intraindividual dynamics of older adults’ perceived balance with objective measures of balance and physical activity. For 30 consecutive days, following a comprehensive fall risk assessment, 20 older adults rated their balance confidence (Activities Balance Confidence scale) at that moment and then performed five standardized balance assessments measured via smartphone accelerometer held to their chest. Physical activity was measured with an activity monitor. Baseline measurements of fall risk differentiated the extent of intraindividual variation and co-variation of balance and physical activity. For some participants, actual and perceived balance became more closely aligned as the study progressed. The implications of the findings for life-span perspectives on aging and fall prevention are discussed.

